# Sifting disease-causing signal from genomic noise

**DOI:** 10.1186/1753-6561-6-S6-O8

**Published:** 2012-10-01

**Authors:** Daniel G MacArthur

**Affiliations:** 1 Analytic and Translational Genetics Unit, Massachusetts General Hospital, Boston, MA 02114, USA; 2 Broad Institute of Harvard and MIT, Cambridge, MA 02142, USA

## 

Recent advances in DNA sequencing technology are transforming our understanding of the genetic basis of rare human diseases. It is now possible to rapidly and cost-effectively interrogate the majority of protein-coding bases in the human genome (known collectively as the exome), finding mutations that would have been difficult if not impossible to discover with the traditional approaches of linkage and candidate gene sequencing.

However, unambiguously identifying the disease-causing mutations in a patient's exome remains challenging. Next-generation sequencing, while powerful, still requires careful filtering to remove errors and is underpowered for discovery of larger insertions/deletions (indels) and complex variants; coverage of genes is incomplete due to biases in DNA capture and sequencing; and predicting the likely functional impact of observed variants is still an immature science. Importantly, existing catalogues both of "normal" variation in reference populations and of reported disease-causing mutations are incomplete and biased. Finally, methods for communicating the results of large-scale sequencing to key target audiences - clinicians, patients and researchers from other fields - remain poorly developed.

In this presentation I describe recent advances in variant-calling from next-generation sequencing technology, and their application to exome data from over 15,000 individuals from multiple different disease-specific studies. Functional annotation of sequence variants across these large samples illustrates the surprising degree of putatively functional genetic variation even in apparently healthy individuals. However, the existence of a very large and accurately called reference panel of exomes provides a powerful resource for interpreting the probability of disease causation for variants observed in rare disease samples. I also discuss new approaches to analyzing and presenting the results from family-based studies of protein-coding mutations in rare disease patients.

